# PCSK9 in vascular smooth muscle cells: biology, pathology, and inhibition to fight atherosclerosis

**DOI:** 10.1016/j.athplu.2025.12.001

**Published:** 2026-03-11

**Authors:** Alessio Amorosi, Mathilde Varret

**Affiliations:** a Scuola Superiore Sant'Anna, Pisa, Italy; b Paris Cité University and Sorbonne Paris Nord University, INSERM UMRS 1148, Laboratory for Vascular Translational Science (LVTS), Paris, France

**Keywords:** Human atherosclerosis, PCSK9, Vascular smooth muscle cells

## Abstract

Atherosclerosis remains the principal cause of cardiovascular morbidity and mortality worldwide, with vascular smooth muscle cells (VSMC) serving as central effectors in plaque initiation, progression, and destabilization. Although originally characterized as a hepatic regulator of LDL receptor degradation and systemic cholesterol homeostasis, PCSK9 is increasingly recognized as a pivotal mediator of vascular pathology. Within the arterial wall, VSMC constitute the predominant extrahepatic source of PCSK9, through which it exerts autocrine and paracrine effects on proliferation, migration, phenotypic plasticity, foam cell formation, oxidative stress, inflammation, and calcification. Collectively, these processes destabilize vascular homeostasis and amplify maladaptive crosstalk with endothelial and immune cells, thereby accelerating atherogenesis. Therapeutic inhibition of PCSK9 provides benefits beyond lipid lowering, reinforcing fibrous cap stability, and dampening inflammatory activity within plaques. While monoclonal antibodies and RNA-based silencing therapies are supported of a growing body of clinical data, recent advances include the development of novel oral PCSK9 inhibitors, among which MK-0616 (Enlicitide) has progressed to phase 3 evaluation. Conversely, genome editing, peptide vaccination, and CAP1-targeted biologics remain at a conceptual or early investigational stage and are still distant from regulatory approval. Yet PCSK9 lives a double life: circulating as a systemic regulator of lipids while acting locally as a driver of vascular pathology. Unraveling this duality through focused research is essential to unlock its full potential in cardiovascular medicine.

## Highlights

• VSMC are the main extrahepatic source of arterial PCSK9.
• PCSK9 disrupts VSMC homeostasis via NF – kB reciprocal activation.
• PCSK9 reprograms VSMC, promoting pro-atherogenic pathways.
• PCSK9 enhances pathological crosstalk with endothelial and immune cells.
• PCSK9 inhibition thickens fibrous caps and stabilizes plaques.

## Graphical abstract

**Figure:**
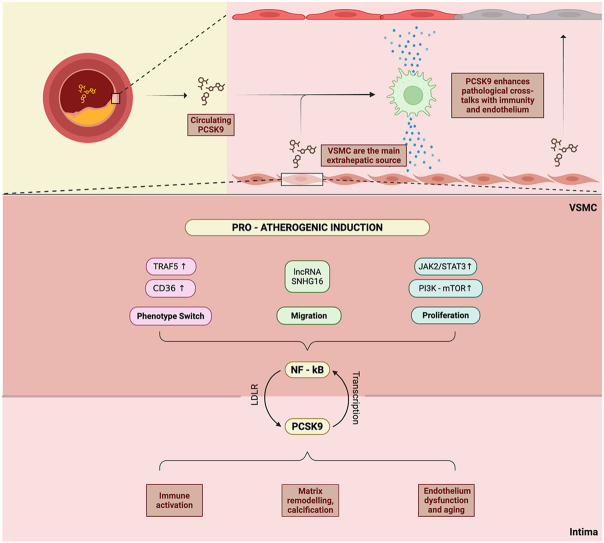

## List of abbreviations

AAA: Abdominal Aortic Aneurysm
ACS: Acute Coronary Syndrome
CD36: Cluster of Differentiation 36
DC: Dendric Cell
EC: Endothelial Cell
ER: Endoplasmic Reticulum
EV: Extracellular Vesicle
IL: Interleukin
LDL-C: Low Density Lipoprotein Cholesterol
LDLR: Low Density Lipoprotein Receptor
LOX-1: Lectin-like OXidized LDL receptor-1
NLRP3: NOD-Like Receptor family, Pyrin domain containing 3
Ox-LDL: Oxidized Low Density Lipoprotein
PCNA: Proliferating Cell Nuclear Antigen
PCSK9: Protein Convertase Subtilisin/Kexin type 9
PDGF: Platelet Derived Grow Factor
ROS: Reactive Oxygen Species
SR-A: Scavenger Receptor Class A
TLR4: Toll – Like Receptor 4
TRAF: TNF Receptor Associated Factor 5
VSMC: Vascular Smooth Muscle Cell
WTAP: Wilms Tumor 1–Associated Protein

## 1 Introduction

Atherosclerotic diseases, including myocardial infarction and stroke, remain the leading cause of death worldwide resulting in roughly 19.8 million deaths each year, representing 32 % of overall mortality in 2022 [https://www.who.int/news-room/fact-sheets/detail/cardiovascular-diseases-(cvds), Last accessed on 2025 Aug 28].

The development of atherosclerosis is driven by a combination of genetic predisposition and environmental influences. In humans, the clinical manifestations typically take several decades to emerge. Well-established risk factors such as hypercholesterolemia, hypertension, diabetes, and smoking play a central role in its pathogenesis [1].

**Fig. 1:**
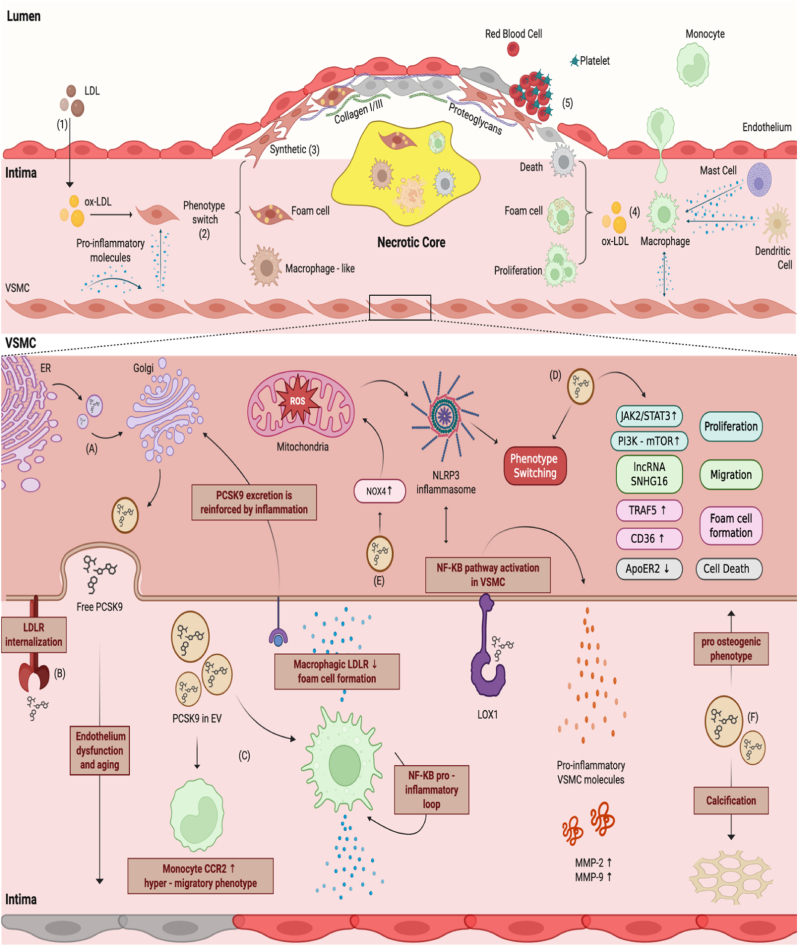
Atherosclerotic development and VSMC PCSK9 pathways. Upper part. (1) Endothelial dysfunction permits LDL entry into the intima, where oxidation generates ox-LDL that VSMC internalize via scavenger receptors such as CD36, LOX-1, and LRP1, triggering a change in phenotype. (2) Macrophage-like cells and foam cells contribute to necrotic-core formation via apoptosis and secondary necrosis, amplifying inflammation through bidirectional crosstalk with VSMC. (3) VSMC derived synthetic cells secrete collagen I/III and proteoglycans, expanding the intima and building the fibrous cap. (4) Subsequential inflammation drives monocyte recruitment and maturation into macrophages through interactions with dendritic cells, mast cells, and bidirectional VSMC signaling. (5) These macrophages become activated, proliferate, and phagocytose ox-LDL, transitioning to foam cells that expand and destabilize the plaque, promoting platelet adhesion/aggregation and red blood cells infiltration. Lower part. (A) In VSMC, PCSK9 is synthesized as a proprotein and matures in the ER, followed by trafficking through the Golgi. PCSK9 is then secreted into the extracellular milieu to act in an autocrine/paracrine fashion, retained to act intracellularly or packaged into extracellular vesicles (EV). (B) Extracellular PCSK9 binds LDLR to promote LDLR endocytosis–lysosomal degradation. (C) Paracrine PCSK9 further degenerates endothelium and reprograms monocytes/macrophages toward pro-inflammatory phenotypes, collectively enlarging and destabilizing the plaque (D) PCSK9 also engages surface signaling (e.g., LOX-1). to trigger NF-κB priming and NLRP3 inflammasome activation, leading to pro-inflammatory molecules (IL-1β/IL-6/CCL2) release and positive feedback. In parallel, PCSK9 activates PI3K–AKT–mTOR and JAK2-STAT3 pathways, enhancing proliferation, migration, and MMP activity while reprogramming epigenetically through lncRNA SNHG16 toward phenotype switch. In late-stage phases, PCSK9 can induce cell death via ApoER2 depletion. (E) PCSK9 further elevates NOX4-dependent ROS, which promotes ox-LDL formation and synthetic switch. (F) PCSK9-enriched EV disseminate pro-inflammatory and pro-calcific cargo (e.g., ALP/annexins/miRNAs), reprogramming neighboring cells and seeding microcalcification. Collectively, these pathways drive neointimal hyperplasia, plaque growth, and instability; PCSK9 inhibition attenuates each of these nodes.

Nevertheless, atherosclerosis is now widely regarded as a chronic and progressive inflammatory disorder of the vascular wall, initiated by the interplay between these risk factors and arterial wall cells. Vascular inflammation of the arterial intima is initiated by the subendothelial retention and oxidative modification of low-density lipoproteins (ox-LDL) (Fig. 1). This process induces endothelial activation, facilitating the recruitment and transendothelial migration of circulating immune cells. Monocytes differentiate into macrophages and endocytose lipids, giving rise to foam cells that contribute to fatty streak formation. Sustained activation of both innate and adaptive immune responses perpetuates a pro-inflammatory microenvironment, promoting vascular smooth muscle cell (VSMC) phenotypic switching, extracellular matrix remodeling, and fibrous plaque development. Over time, these processes undermine vascular integrity and increase susceptibility to plaque rupture and thrombosis, thereby elevating the risk of acute ischemic events [2].

Protein Convertase subtilisin/kexin type 9 (PCSK9) is a 692-aa serine protease, coded in 1p32.3 locus and secreted after an autocatalytic cleavage VFAQ152↓ of its N-terminal side in the endoplasmic reticulum (ER). Subsequently, it remains non-covalently bound to its prodomain, suggesting single-use catalytic activity and a non-enzymatic mode of action. Cellular or plasmatic PCSK9 was firstly discovered for directing the LDL receptor (LDLR) toward lysosomal degradation in pancreas, small intestine and liver, regulating circulating LDL-Cholesterol (LDL-C) level [3].

In VSMCs, the transcriptional regulation of PCSK9 involves both lipid-sensitive and inflammatory pathways, reflecting a complex interplay between metabolic and vascular signaling. The SREBP2–HNF1α axis constitutes a principal mechanism driving PCSK9 transcription under conditions of cholesterol depletion or statin exposure [4]. Whereas pro-inflammatory stimuli and oxidative stress enhance PCSK9 expression through NF-κB–dependent promoter activation. Notably, this regulatory profile mirrors that of LOX-1 (lectin-like oxidized LDL receptor-1), LDLR, and related members of the LDL receptor family, which are similarly induced in response to oxidized lipoproteins and vascular injury [5].

PCSK9 is a well-recognized player in atherosclerosis due to its effect on plasma cholesterol homeostasis, the therapeutic inhibition of which reduces the risk of cardiovascular disease [[6], [7], [8]]. Regardless of that, PCSK9 is increasingly recognized for its major role in vasal homeostasis and atherogenesis. In blood arteries, VSMC are among the primary expressers of this protein, where it functions as a pro-inflammatory mediators [9] (Fig. 1). Interestingly, in addition to its contribution to atherogenesis, ectopic expression of PCSK9 by vascular smooth muscle cells has also been shown to promote aortic dissection [10], highlighting its broader impact on vascular wall stability and homeostasis.

Beyond reducing LDL-C, PCSK9 inhibition could reduce atherosclerosis, particularly in patients who have already reached their target levels or who do not have dyslipidemia, highlighting its therapeutic and a promising direction in cardiovascular research. In this review, we aim to provide an overview of the data on the effect of PCSK9 in VSMC biology and its consequences on the progression of atherosclerosis.

## 2 Methods

For this state-of-the-art review, the literature search was performed by the PubMed database, considering all manuscripts that meet the following search criteria: (PCSK9 [Title]) AND (smooth muscle cells [Title/Abstract]) AND (English [Language], done the May 05, 2025. Through the analysis of the 33 references from this search, we selected all of the studies cited in this review.

## 3 PCSK9 expression in the arterial wall

PCSK9, a regulator of vascular homeostasis and atherogenesis, shows cell-type–specific expression in the arterial wall (Table 1). In coronary scRNA-seq datasets signal is most consistent in VSMC clusters, while endothelial and macrophage clusters display low or intermittent detection unless inflamed or stressed [11]. Given PCSK9's low extrahepatic abundance and the limited sensitivity of droplet scRNA-seq, pseudobulk aggregation with orthogonal validation (RNAscope, IHC, sorted bulk RNA or spatial transcriptomics) is the preferred approach to define cellular sources.

**Table 1:** Different expression of PCSK9 in the main cellular protagonist of atherosclerosis.

Cell type	Baseline PCSK9 (mRNA/protein)	Induced PCSK9 (by stress/inflammation)	How to interpret
Endothelium (ECs) [5,[12], [13], [14]]	Very low/undetectable at rest in primary human ECs.	↑ with oxLDL, TNF-α, LPS; disturbed/low shear (ROS) models show endothelial PCSK9 upregulation.	ECs are not a major source at baseline but become PCSK9-positive in pro-atherogenic contexts.
Vascular smooth muscle cells (VSMC) [[15], [16], [17], [18]]	Moderate–high basal expression; active secretion in human VSMC.	↑ with oxLDL, cytokines (TNF-α), PDGF; metabolic stress (high glucose/insulin resistance).	Principal extra-hepatic source within the wall; autocrine/paracrine PCSK9 modulates phenotype and neighbors.
Monocytes [15,19,20]	Essentially none/very low in circulating human monocytes (scRNA: HPA single cell).	Primarily respond to extracellular PCSK9 (adhesion/chemotaxis phenotypes); endogenous induction remains limited in undifferentiated monocytes.	Consider monocytes targets/responders rather than sources under most human conditions.
Macrophages [15,19,20]	Low at rest in primary human macrophages; can accumulate exogenous PCSK9 via LDLR.	↑ with oxLDL (THP-1–derived macrophages show dose-dependent PCSK9 mRNA/protein); inflammatory plaque milieu increases lesional PCSK9.	In lesions, macrophages both express (when stimulated) and uptake PCSK9, amplifying inflammatory signaling.
Dendritic cells (DCs) [21]	Low at baseline.	↑ with oxLDL; PCSK9 knockdown blunts DC maturation and T-cell activation.	Activated DCs become a PCSK9 source that primes T-cell inflammation.
T lymphocytes [21]	Minimal/none (not a recognized PCSK9 source).	No robust evidence of inducible production; T-cell activation is modulated indirectly by DC/SMC PCSK9.	Treat T cells as targets, not sources, in plaque immunology.
Mast cells	Insufficient evidence for endogenous production.	–	Participate in inflammation, but PCSK9 production not established.
Neutrophils	Unclear/likely minimal as producers.	–	PCSK9 can modulate neutrophil activity systemically; production by neutrophils not established.
Platelets (anuclear) [[22], [23], [24]]	Protein detectable; no mRNA synthesis (anucleate cells store PCSK9 acquired from plasma or megakaryocytes).	Release of stored PCSK9 upon activation (e.g., by collagen, thrombin, or LDL exposure); amplified under inflammatory or pro-thrombotic conditions.	Platelets act as a reservoir and amplifier of circulating PCSK9, contributing to platelet activation, thrombus formation, and vascular inflammation rather than de novo synthesis.

Healthy endothelial cells (EC) have little to no basal PCSK9 expression. In line with this, no significant PCSK9 protein secretion occurs from EC under resting condition [12]. However, inflammatory stimuli can induce PCSK9 in endothelium. The exposure to ox-LDL or TNF-α markedly upregulates PCSK9 expression in EC, in an in vitro model of arterial inflammation [5,13]. Likewise, a hemodynamic study showed that in mouse aortic **areas of disturbed flow (low shear stress) provoke increased PCSK9 expression in EC (and VSMC) via reactive oxygen species (ROS) signaling** [14]**.**

**In the arterial wall, the prime extrahepatic source of PCSK9 are VSMC, where the protein is expressed and biologically active in baseline conditions** [12]**.** Inflammation further heighten VSMC-PCSK9 production. For example, **ox-LDL and TNF-α or low shear stress exposure significantly increased PCSK9 expression in cultured VSMC** [5,14]. Contextually, there is attested no signific changes in cytokine receptors induced by PCSK9 [25]. Recent work also demonstrates a metabolic–PCSK9 axis in VSMC: **aortic VSMC from obese, insulin-resistant rats** exhibit elevated basal *Pcsk9* mRNA/protein versus lean littermates, and **high glucose further induces** *Pcsk9* effects. These data support a **positive association** between hyperglycemia/obesity and VSMC-PCSK9 expression [15,16]. Importantly, PCSK9 is present within atherosclerotic plaques, largely colocalized with VSMC [17].

Circulating monocytes and tissue macrophages exhibit minimal intrinsic PCSK9 expression in healthy and basal conditions [12]. Interestingly, Jaén et al., 2022 [18] corroborated that human macrophages do not express PCSK9 themselves, but instead readily internalize extracellular PCSK9 via LDL receptors, favoring a dysfunctional phenotype. Under atherogenic stress, ox-LDL engagement of lectin-like oxidized LDL receptor-1 (LOX-1) and cluster of differentiation 36 (CD36) activates NF-κB, leading to a marked induction of PCSK9 in macrophages [19].

Dendritic cells (DC), key antigen-presenting cells in atherosclerosis, express PCSK9 under disease-relevant conditions; in human monocyte-derived DC, exposure to ox-LDL **upregulates** PCSK9, TNF-a, IL-1B and IL-6 expression (via NF-kB) [20].

In the same experiment, T-cell (with no basal expression of PCSK9) in co-culture **became highly activated (proliferating and secreting IFN-γ/IL-17)**, skewing toward Th1/Th17 phenotypes. Crucially, **blocking PCSK9 in the DC blunted this T-cell activation** and instead promoted regulatory T-cell features. This suggests that **PCSK9, produced by DCs or other cells, can modulate T-cell activity** in the atherosclerotic environment [20].

These expression patterns support PCSK9-driven autocrine/paracrine loops in plaque remodeling and underscore the need for further confirmation analysis.

## 4 PCSK9 effects on VSMC biology

### 4.1 Migration

**Fig. 2:**
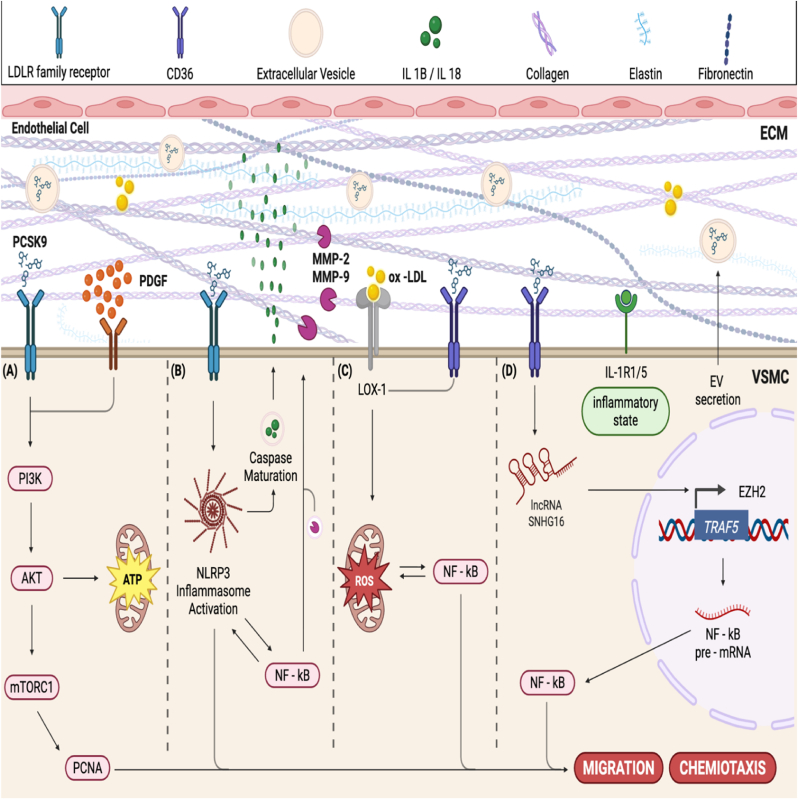
PCSK9 pro-migratory influence on VSMC. Note that PCSK9 can be found extracellularly in EV or floating freely.

Accumulating evidence indicates that PCSK9 exerts a pro-migratory influence on VSMC through diverse molecular cascades, thereby facilitating vascular remodeling processes central to atherogenesis [21] (Fig. 2). Early murine work first linked PCSK9 to VSMC motility: *Pcsk9* knockout markedly curtailed migration and was not accompanied by filopodial remodeling, which is characteristic of a motile phenotype [26].

Mechanistically, PCSK9 **indirectly activates** the PI3K–AKT–mTOR pathway (Fig. 2A) in VSMC also by amplifying upstream receptor signaling for chemiotaxis (e.g., PDGF–PDGFR), leading to increased Akt/mTOR phosphorylation and promoting proliferative and migratory behavior, with concomitant rises in proliferating cell nuclear antigen (PCNA) [27,28] (Fig. 2A).

An additional process, delineated in mice models of allograft vasculopathy, is **PCSK9-mediated activation of the** NOD-like receptor family, pyrin domain containing 3 (**NLRP3) inflammasome (**Fig. 2B**)**, which potentiates vascular inflammation and augments VSMC motility [29]. Downstream of PCSK9, activation of the NLRP3 inflammasome drives caspase-1–dependent maturation of IL-1β and IL-18, which act in autocrine and paracrine fashion to enhance VSMC chemotaxis and pericellular matrix remodeling. These cytokines induce (and activate) the gelatinases MMP-2 and MMP-9 (via NF-κB and MT1-MMP) thereby accelerating extracellular matrix degradation and facilitating VSMC migration [30].

PCSK9 also acts as a pro-inflammatory agonist in endothelium and macrophagic cells, directly engaging Toll-like receptor 4 (TLR4) and activating NF-κB signaling (Fig. 2C), which in turn upregulates cytokines (IL-1β, TNF-α, MCP-1) and migration, while creating an inflammatory environment with induction of matrix-degrading enzymes [31,32]. Given that VSMC express functional TLR4, and NF-KB pathway is generally active, a similar mechanism is plausible but requires direct validation. NF-κB–dependent upregulation of PCSK9 in VSMC amplifies LOX-1 expression; LOX-1 ligation boosts ROS and re-engages NF-κB, inducing MMP-2/-9 and other remodeling proteins that facilitate VSMC migration and the synthetic phenotype [5].

Emerging evidence indicates that PCSK9 modulates VSMC migration also through non-coding RNA–mediated gene regulation. In VSMC, PCSK9 upregulates the lncRNA SNHG16 (Fig. 2D), which acts as a scaffold for the histone methyltransferase EZH2 and recruits it to the *TRAF5* gene promoter, depositing repressive H3K27me3 marks. Epigenetic silencing of TRAF5— encoding an adaptor that normally constrains TNF/NF-κB signaling—removes this brake, thereby enhancing NF-κB activity and promoting VSMC proliferation and migration. Collectively, the PCSK9–SNHG16/EZH2–TRAF5 axis introduces an epigenetic layer to the control of VSMC phenotypic switching and motility [33].

Taken together, the literature positions PCSK9 as a significant regulator of VSMC migratory capacity with direct implications for plaque growth and vascular remodeling.

### 4.2 Proliferation and cell death

**Fig. 3:**
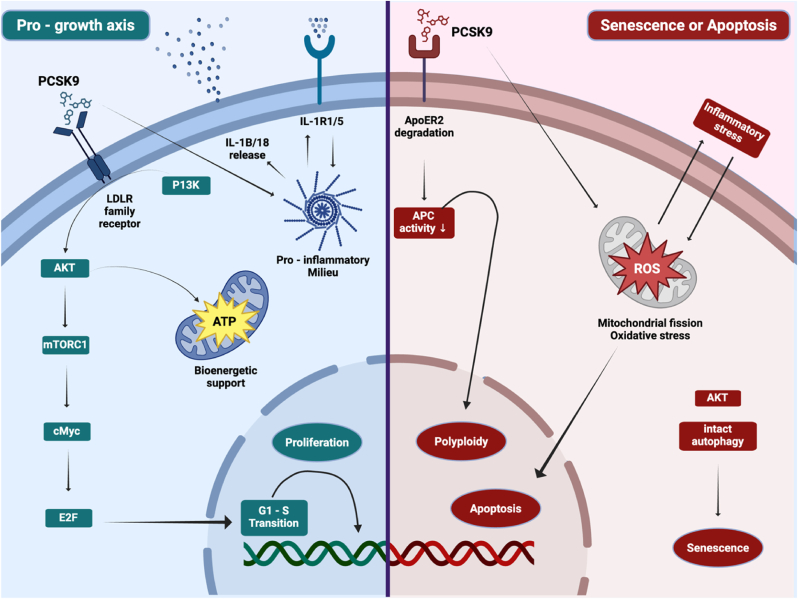
PCSK9 shapes VSMC destiny in a context dependent way.

PCSK9 shapes VSMC fate in a context-dependent way. During vascular injury/inflammatory settings it favors a synthetic, proliferative program and neointimal expansion, whereas PCSK9 persistent expression in human VSMC and vulnerable plaques is linked to growth arrest, senescence, and apoptosis (Fig. 3).

PCSK9, analogous to its pro-migratory role, engages the PI3K–AKT–mTOR axis in VSMC to enforce phenotype switch and promote lesion building (Fig. 3 left section). Downstream, mTORC1-dependent phosphorylation of S6K and 4E-BP1 elevates Cyclin-D1/c-Myc, while AKT inactivates FOXO factors and permits CDK4/6-mediated Rb phosphorylation with E2F-driven G1–S progression. In parallel, AKT provides pro-survival and metabolic support (enhanced glucose uptake, glycolysis, and macromolecular biosynthesis), supplying the bioenergetic substrate for clonal VSMC expansion [27,34]. This pro-growth cascade is also amplified by PCSK9-driven activation of the NLRP3 inflammasome and the resulting inflammatory cytokines (pyroptosis is plausible but still not confirmed) [29].

Additionally, PCSK9 can promote degenerative VSMC fates by depleting ApoER2 and provoking polyploidy with senescence or apoptosis (Fig. 3 right section). Mechanistically, ApoER2 scaffolds PP2A-C to CDC20 to activate the APC/C; its loss disrupts anaphase progression and causes cytokinesis failure. The terminal outcome is context dependent: chronic, partial ApoER2 loss under adequate trophic/pro-survival support (e.g., intact AKT/mTOR signaling and autophagy) stabilizes senescent arrest, whereas abrupt or pronounced loss—particularly in oxidative or inflammatory environments—drives mitochondrial dysfunction and caspase-dependent cell death [[35], [36], [37]].

Another way that PCSK9 induces apoptosis in VSMC consists in mitochondrial fission signaling through p38 MAPK–DRP1: p38-dependent DRP1 phosphorylation promotes its recruitment to MFF/Fis1, driving excessive fission. The fragmented network exhibits dissipation of the mitochondrial membrane potential, ROS accumulation with mtDNA lesions, and OPA1-mediated cristae remodeling, which licenses BAX/BAK-dependent outer-membrane permeabilization, caspase-9/-3 activation, and apoptosis [38]. In parallel, oxidized or mislocalized mtDNA generated by this dysfunction is detected by canonical DNA-sensing pathways, activating NF-κB which upregulates PCSK9 transcription. Subsequently, this feed-forward circuit escalate in VSMC loss and promotes plaque destabilization [39].

Beyond its pro-apoptotic actions, PCSK9 also modulates autophagy in VSMCs, thereby influencing proliferative and remodeling responses. In carotid-ligated arteries and PDGF-BB–stimulated VSMC cultures, PCSK9 expression increases in parallel with autophagy markers, including Beclin-1 upregulation, LC3-I to LC3-II conversion, and p62 degradation, consistent with enhanced autophagic flux. Conversely, genetic deletion or silencing of PCSK9, as well as pharmacological inhibition by evolocumab, markedly reduces autophagy-related protein turnover and cell proliferation, suggesting that PCSK9 acts as a positive regulator of autophagic machinery in proliferating VSMCs. Mechanistically, PCSK9 appears to promote PDGF-driven autophagy via ROS generation and activation of MAPK and NF-κB pathways, linking metabolic stress responses to structural remodeling of the vascular wall. These findings suggest PCSK9 supports VSMC survival and neointimal formation through autophagy-dependent mechanisms, extending its functional impact beyond lipid metabolism [27].

In the context of abdominal aortic aneurysm (AAA), WTAP (Wilms tumor 1–associated protein) was shown to regulate VSMC homeostasis through analyses of patient-derived tissues and angiotensin II–stimulated human aortic smooth muscle cells, where its expression was markedly upregulated under pathological conditions. Mechanistically, WTAP enhanced the stability of PCSK9 transcripts through an N6-methyladenosine (m6A) dependent interaction with the Insulin-like growth factor 2 mRNA-binding protein 2 (IGF2BP2), thereby sustaining elevated PCSK9 expression. Functionally, this WTAP–PCSK9 axis promoted VSMC senescence, apoptosis, ferroptosis, and pro-inflammatory signaling, while silencing of WTAP reduced PCSK9 levels and conferred protection against these maladaptive responses. These findings delineate a potential novel epi-transcriptomic pathway that remains to be confirmed by further studies [40].

Therefore, future work should also define the quantitative microenvironmental thresholds and receptor–trafficking determinants of VSMC destiny, resolve NLRP3–mTOR–autophagy crosstalk at single-cell and spatial resolution, confirm pyroapoptotic death and test temporally and cell-type–targeted PCSK9 modulation to stabilize human atherosclerotic lesions.

### 4.3 Scavenger receptor boost and foam cell formation

In VSMC, PCSK9 contributes to foam‐cell formation through multiple LDLR‐independent pathways, like upregulation of scavenger receptors, modulation of cholesterol efflux, and activation of inflammatory/epigenetic signaling.

Foam cell formation depends on scavenger receptors (pattern-recognition receptors mediating the uptake of modified lipoproteins) among LOX-1, CD36, and scavenger receptor class A (SR-A) are central in VSMC and macrophages [41].

Recent findings suggest that PCSK9 and LOX-1 may participate in a reciprocal regulatory loop in VSMC, whereby each could promote the other's expression and pro-inflammatory activity. Mechanistically, ox-LDL or inflammatory stimuli activate NF-κB and ROS signaling, leading to LOX-1 upregulation, which has been shown to enhance PCSK9 expression in vascular cells [5]. PCSK9, in turn, induces LOX-1 transcription, potentially sustaining a positive feedback cycle that amplifies ox-LDL uptake, inflammatory cytokine production, and VSMC phenotypic switching toward a foam-cell–like state. This crosstalk, although more firmly established in endothelial cells and macrophages, is increasingly implicated in VSMC as a contributor to maladaptive vascular remodeling, thereby linking the PCSK9–LOX-1 axis to atheroma progression and plaque destabilization [42].

CD36 is a key ox-LDL receptor in VSMC, yet the role of PCSK9 in regulating CD36 has not been directly investigated in this cell type [43]. Evidence from other systems suggests both upregulation (macrophages) and lysosomal degradation (liver), leaving its impact in VSMC unresolved and likely dependent on alternative receptors such as LOX-1 [44].

It is also plausible that, similarly to macrophages, PCSK9 upregulates SR‐A in VSMC under inflammatory conditions. No studies have yet shown PCSK9 directly degrading SR‐A as it does LDLR, so SR‐A may remain available to drive foam formation under PCSK9‐rich conditions [44].

For cholesterol outflow, PCSK9 suppresses ABCA1 expression in macrophages and it is reasonable to hypothesize that an analogous regulatory mechanism may exist in VSMC where inhibition of ABCA1 could further compromise efflux capacity and promote foam cell formation; however, this possibility has not yet been experimentally established [45].

Beyond its effects on scavenger receptors, PCSK9 can also modulate VSMC lipid managing through epigenetic mechanisms. Recent work demonstrated that PCSK9 promotes foam cell formation via lncRNA SNHG16 – EZH2 – H3K27me3 axis (described in 2.1 section). This epigenetic modification enhances the transcription of tumor necrosis factor receptor–associated factor 5 (TRAF5), an adaptor molecule implicated in NF-κB signaling and inflammatory activation. Increased TRAF5 expression, in turn, drives pro-inflammatory gene expression and increases ox-LDL uptake by VSMC, thereby accelerating their transition into foam cells [33].

There may also be an inflammatory contribution: PCSK9 binding to CAP1 and activating the Syk/PKCδ/NF-κB pathway, shown in macrophages and endothelial cells, could plausibly occur in VSMC, promoting cytokine release, TLR4 signaling, and scavenger receptor expression, thereby enhancing ox-LDL uptake and foam cell formation [46].

Thus, future research is required to delineate the specific mechanisms through which PCSK9 promotes foam cell formation in VSMC, encompassing its potential regulation of scavenger receptors, cholesterol efflux transporters, epigenetic modulators, and inflammatory cascades.

### 4.4 Phenotype switch

PCSK9 functions as a critical modulator of VSMC phenotypic plasticity, promoting the transition from a contractile to a synthetic, proliferative, inflammatory, or osteogenic state, thereby facilitating vascular remodeling processes that underlie atherogenesis.

As seen in 2.2 section, overexpression of PCSK9 in cultured aortic VSMC caused a marked decrease in ApoER2, triggering DNA synthesis arrest, polyploidization and ultimately cell senescence and apoptosis toward a degenerative synthetic – like state [35].

PCSK9 also promotes the acquisition of a synthetic and inflammatory phenotype in human VSMC by upregulating scavenger receptor pathways. Exposure to PCSK9 enhances LOX-1 and VCAM-1 expression, thereby increasing ox-LDL uptake and stimulating the secretion of pro-inflammatory cytokines, features characteristic of VSMC dedifferentiation and vascular inflammation [5]. As part of the inflammatory and degenerative reprogramming, enforced overexpression of human PCSK9 in cultured VSMC induces a loss of the contractile phenotype. Greco et al., 2022 [47] demonstrated that PCSK9-overproducing VSMC shift from an elongated, α-actin–rich morphology to a rounded, α-actin–deficient state, characterized by accelerated proliferation and a marked downregulation of contractile markers such as ACTA2 and calponin.

Recent studies have shown that PCSK9 overexpression can also drive VSMC toward an osteogenic, pro-calcific phenotype. Under high-phosphate conditions mimicking uremic calcification, PCSK9 markedly increased calcium deposition, coupled with enhanced release of extracellular vesicles (EV) enriched in calcium and alkaline phosphatase, upregulation of osteogenic markers (BMP2, ALP), and downregulation of calcification inhibitors (matrix GLA protein, osteopontin) [48].

In addition to experimentally established pathways, several novel mechanisms (largely derived from studies in rat VSMC) have been proposed to explain how PCSK9 may contribute to the contractile-to-synthetic phenotype switch. One hypothesis is that PCSK9, by promoting LDLR degradation, alters membrane cholesterol content and disrupts lipid-raft organization, thereby modifying membrane fluidity and receptor signaling in a way that favors proliferative and dedifferentiated states [48].

A second mechanism involves enhancement of the mevalonate pathway: PCSK9 upregulation has been linked to increased HMG CoA reductase activity and accumulation of isoprenoid intermediates, which sustain prenylation of small GTPases (Ras, Rho, Rac) known to orchestrate cytoskeletal remodeling and cell cycle progression during VSMC dedifferentiation [48]. Finally, inflammatory receptor pathways such as TLR4/NFκB have been suggested, based on evidence from macrophages and the observed PCSK9–LOX-1 feed-forward loop, which could propagate pro-inflammatory signaling and reinforce the acquisition of synthetic and degenerative features [27].

While still speculative and not directly demonstrated in human VSMC, these mechanisms provide plausible extensions of PCSK9's influence on vascular cell plasticity beyond its canonical role in lipoprotein metabolism. All in all, future research should aim to delineate the precise intracellular signaling networks by which PCSK9 governs VSMC phenotype switching, with particular focus on distinguishing canonical lipid-regulatory effects from direct cell-intrinsic pathways.

## 5 Direct roles of PCSK9 in inflammation

PCSK9 is now recognized to promote vascular inflammation through multiple LDLR-independent mechanisms [49,50]. In human atherosclerosis, higher circulating PCSK9 correlates with inflammatory markers and lesion burden [47,51]. Using a human vascular microphysiological system, researchers further demonstrated that PCSK9 activation promotes early atherosclerotic changes by enhancing endothelial inflammation and monocyte adhesion [52].

A representative example of a VSMC pro-inflammatory pathway is the PCSK9–LOX-1 axis. In this context, PCSK9 induces LOX-1 upregulation, with reciprocal reinforcement of PCSK9 expression by LOX-1 activation [5]. The engagement of the pathway facilitates ox-LDL internalization and elicits the secretion of pro-inflammatory cytokines via NF-κB activation, including IL-6 and TNF-α, alongside the induction of adhesion molecules such as VCAM-1, thereby sustaining inflammation and promoting leukocyte recruitment [46].

A second example of a PCSK9-driven pro-inflammatory cascade in VSMC is the activation of the NLRP3 inflammasome. Mechanistically, PCSK9-induced ROS and NF-κB signaling provide the priming signal, while caspase-1 activation drives inflammasome assembly, cytokine maturation, and potentially pyroptosis. The downstream release of IL-1β and IL-18 amplifies local vascular inflammation, in a reciprocal loop [29].

A final example of a PCSK9-mediated pro-inflammatory pathway is its transfer through extracellular vesicles (EV) secreted by VSMC. In human VSMC, PCSK9 overexpression enriches EV cargo with PCSK9 protein together with adhesion molecules, lipids, and nucleic acids. When released, these EV activate endothelial cells by upregulating adhesion molecule expression and stimulate monocytes to secrete inflammatory cytokines such as IL-1β, IL-6, and TNF [47].

Nonetheless, PCSK9 activity needs to be contextualized. A recent study demonstrated that antibody-mediated neutralization, while beneficial in chronic atherosclerosis, exerts detrimental effects following bare-metal stent implantation in mice. In this acute injury setting, PCSK9 inhibition aggravated neointimal hyperplasia, enhanced inflammatory cell infiltration, and impaired re-endothelialization, suggesting that endogenous PCSK9 may contribute to vascular repair processes. These findings highlight the dual and situation-specific roles of PCSK9, underscoring the need for careful consideration of therapeutic timing and context when employing PCSK9 inhibitors in cardiovascular interventions [53].

PCSK9 also participates in a local pro-inflammatory circuit with both autocrine and paracrine dimensions. Autocrinely, oxidant and biomechanical stress upregulate PCSK9 via ROS- and NF-κB–dependent signaling, which in turn reinforces a synthetic/pro-inflammatory VSMC phenotype and increases adhesion molecule expression, favoring leukocyte docking and matrix remodeling [15,29]. Paracrinely, PCSK9 engages the LOX-1 axis, where PCSK9 and LOX-1 reciprocally amplify each other in response to oxLDL, augmenting cytokine release and lipid uptake in the vascular wall [5]. PCSK9 also packages into VSMC-derived extracellular vesicles, which activate neighboring endothelial cells and monocytes/macrophages, propagating inflammatory signaling and promoting lesion progression [47]. Collectively, these mechanisms position VSMC-derived PCSK9 as a driver of vascular inflammation and remodeling beyond systemic lipid control, but further research is required to elucidate the precise molecular pathways, cellular interactions, and contextual factors. Emerging studies also indicate that PCSK9 contributes to the pathogenesis of abdominal aortic aneurysm (AAA). Increased PCSK9 activity promotes inflammation, extracellular matrix degradation, and VSMC damage, collectively weakening the aortic wall and facilitating aneurysm formation and progression [54]. Beyond these demonstrated pathways, other processes remain largely inferential: in immune cells, PCSK9 can signal through TLR4 to activate NF-κB, and CAP1 has been identified as a receptor mediating PCSK9-induced cytokine production via Syk/PKCδ-dependent pathways [32,46]. Although both TLR4-and CAP1-mediated signaling are biologically plausible in VSMC, direct evidence is currently lacking. Taken together, these findings suggest that future research should prioritize defining the relative contribution of validated pathways in human VSMC while experimentally testing CAP1- and TLR4-dependent signaling in VSMC establish their relevance in atherosclerosis.

### 5.1 Interaction with other cell types during atherogenesis

There is growing evidence that PCSK9 secreted by VSMC establishes multiple pro-inflammatory interactions with vascular and immune cells during atherogenesis, thereby amplifying local pathophysiological circuits [[55], [56], [57]] (Table 2).

**Table 2:** PCSK9 effects in the main cellular protagonist of atherosclerosis.

Cell Type	Adhesion molecules/Chemotaxis	Lipid Handling	Inflammatory Pathways	Survival/Apoptosis	Interpretation
Endothelial cells [32,46,50,58]	↑ VCAM-1, ICAM-1, E-selectin (NF-κB, LOX-1 mediated)	↑ LDL permeability; ↑ ox-LDL uptake	↑ NF-κB activation; ↑ ROS; defective efferocytosis	↑ Senescence; ↑ apoptosis (Bax, Caspase-3/9); impaired nitric oxide bioavailability	PCSK9 promotes endothelial dysfunction, inflammation, and barrier disruption.
Monocytes [50,59]	↓ CCR2, altered chemokine receptor profile	↓ LDLR; reduced lipid uptake	↑ NF-κB–dependent cytokines (IL-1β, TNF-α, IL-6)	Priming toward inflammatory phenotype	PCSK9 shifts monocytes toward pro-inflammatory, foam cell–prone phenotype.
Macrophages [12,46,60]	↑ Adhesion via LOX-1, CD36; enhanced oxLDL uptake	↓ LDLR, ↓ ABCA1/ABCG1; ↑ LOX-1/CD36 foam cell formation	↑ NF-κB activation; ↑ NLRP3 inflammasome; ↑ IL-1β; ↑ ROS	Foam cell transition and metabolic stress	PCSK9 sustains macrophage-driven inflammation and lipid accumulation.
Dendritic cells [20]	↑ Co-stimulatory molecules; enhanced T-cell priming	Indirect – promotes lipid antigen presentation	↑ Cytokine release; ↑ antigen presentation capacity	–	PCSK9 potentiates dendritic cell–mediated adaptive immune activation.
T lymphocytes [20]	Enhanced priming and polarization (Th1/Th17)	Indirect – modulated via antigen exposure	↑ Pro-inflammatory cytokines	↑ Survival modulation; altered effector functions	PCSK9 supports adaptive immune polarization toward inflammatory phenotypes.
Platelets [[22], [23], [24]]	↑ Activation and aggregation (via CD36, ROS/NF-κB)	Not directly lipid-related	↑ ROS/NF-κB signaling; thrombo-inflammation	↑ Platelet survival and hyper-reactivity	PCSK9 enhances platelet-driven thrombosis and vascular inflammation

For instance, Greco et al., 2022 [47] demonstrated that VSMC-PCSK9 released within EV activates endothelial cells, triggering NF-κB signaling and driving the upregulation of adhesion molecules such as VCAM-1 and ICAM-1. This activation is reinforced by PCSK9-mediated induction of LOX-1 and the generation of ROS, which together enhance ox-LDL uptake, sustain NF-κB activation, and amplify the inflammatory endothelial response [5]. Beyond adhesion molecule expression, PCSK9 promotes endothelial degeneration and premature senescence, processes linked to mitochondrial dysfunction, impaired nitric oxide bioavailability, and regulation of the SIRT1 pathway [58]. In parallel, PCSK9 favors endothelial apoptosis, as knockdown experiments demonstrated reduced expression of pro-apoptotic mediators such as Bax, Caspase-3, and Caspase-9, together with increased Bcl-2, underscoring its role in cell survival control [61]. Furthermore, PCSK9 has been implicated in defective efferocytosis by endothelial cells, thereby limiting the clearance of apoptotic bodies and contributing to vascular aging [59]. Collectively, these mechanisms converge to enhance leukocyte adhesion, lipoprotein transcytosis, and endothelial dysfunction, sustaining the chronic inflammatory environment characteristic of atherosclerotic lesions.

Following its effects on endothelial cells, VSMC PCSK9 also modulates monocyte function. Experimental data demonstrate that PCSK9 released from VSMC reduces LDLR expression on monocytes and concomitantly downregulates CCR2, a chemokine receptor critical for CCL2-driven migration [60]. Beyond these effects, PCSK9 promotes a pro-inflammatory monocyte phenotype by enhancing NF-κB dependent secretion of cytokines such as IL-1β, TNF-α, and IL-6, and by upregulating scavenger receptors including LOX-1 and CD36, thereby fostering ox-LDL uptake and foam cell transition [46]. Additional evidence suggests that PCSK9 may alter monocyte chemokine receptor profiles, and prime their differentiation toward an inflammatory macrophage phenotype [47]. Through these combined mechanisms, VSMC PCSK9 (whether secreted as a soluble protein or conveyed via extracellular vesicles) diminishes monocyte lipid uptake, attenuates chemotactic responses, and sustains pro-inflammatory activation.

VSMC PCSK9 also exerts direct effects on macrophages. Among vascular cells, PCSK9 is produced by VSMC but not by macrophages or endothelial cells, and conditioned media from VSMC has been shown to reduce LDLR expression in human macrophages, thereby impairing receptor-mediated lipid clearance and favoring cholesterol accumulation [12]. Beyond LDLR regulation, PCSK9 decreases the expression of cholesterol efflux transporters ABCA1 and ABCG1, while simultaneously upregulating scavenger receptors such as LOX-1 and CD36, reinforcing lipid loading and foam cell formation [45]. In parallel, VSMC extracellular vesicles enriched in PCSK9 promote macrophage activation by inducing pro-inflammatory cytokine production and enhancing ox-LDL uptake, contributing to an atherogenic phenotype [47].

Beyond its effects on endothelial cells, monocytes, and macrophages, VSMC PCSK9 may also influence other immune and vascular effector cells. Experimental data suggest that PCSK9 enhances dendritic cell activation by promoting antigen-presenting capacity and pro-inflammatory cytokine release, thereby facilitating T-lymphocyte priming and polarization toward a Th1/Th17 phenotype. In parallel, PCSK9 has been implicated in modulating T-cell survival and effector functions, favoring a pro-inflammatory milieu within atherosclerotic lesions [48]. Concerning platelets, PCSK9 released in the vascular microenvironment promotes platelet activation and aggregation through CD36 engagement and subsequent ROS/NF-κB signaling, thereby contributing to thrombo-inflammatory processes [22]. Increasing evidence indicates that platelets themselves represent an additional source of PCSK9. In patients with coronary artery disease, platelet count and reactivity correlate positively with circulating LDL-cholesterol levels, linking platelet activation to lipid metabolism [23]. Experimental studies have shown that activated platelets express and release PCSK9, particularly in response to LDL, and that this release enhances thrombus formation, monocyte recruitment, and macrophage differentiation—effects mitigated by PCSK9 inhibition. Immunohistochemical analyses have localized PCSK9 to platelet- and macrophage-rich regions within atherosclerotic plaques [24].

Overall, current evidence positions VSMC PCSK9 as a central mediator of vascular inflammation through multifaceted interactions with endothelial, immune, and platelet compartments. Yet, many of these effects have not been investigated using PCSK9 directly produced by VSMC, and their attribution relies on the plausible inference that soluble or EV PCSK9 released by VSMC exerts similar actions. Future studies should delineate the relative weight of these pathways in human atherosclerosis.

### 5.2 ROS generation

PCSK9 promotes oxidative stress in VSMC by enhancing intracellular ROS production, which destabilizes cellular homeostasis and contributes to inflammatory signaling, senescence, and cell death [15]. This pro-oxidant activity is increasingly recognized as a key driver of vascular dysfunction and atherogenesis [62].

For example, PCSK9 overexpression activates p38 MAPK, which phosphorylates Drp1 and drives its recruitment to the mitochondrial outer membrane, leading to excessive fission and membrane depolarization [38]. This disruption of the electron transport chain, particularly at complexes I and III, enhances electron leakage and promotes the formation of superoxide anions (O_2_•−), which are subsequently converted by SOD2 into hydrogen peroxide (H_2_O_2_). Accumulated H_2_O_2_, in the presence of free iron, generates highly reactive hydroxyl radicals (•OH), thereby amplifying oxidative damage to mitochondrial DNA and proteins [39]. These redox alterations further compromise respiratory chain function, establishing a feed-forward loop of ROS overproduction that sustains oxidative stress. In turn, ROS-mediated mitochondrial injury promotes the release of cytochrome *c* and activation of caspase-9, while simultaneously shifting the balance of Bcl-2 family proteins toward pro-apoptotic members, thereby reinforcing mitochondrial permeabilization, triggering the caspase cascade, and driving VSMC apoptosis that contributes to plaque vulnerability [38].

Another key circuit is represented by the LOX-1–NADPH oxidase axis, through which PCSK9 indirectly augments oxidative stress in VSMC [5]. By upregulating LOX-1 expression, PCSK9 increases cellular sensitivity to ox-LDL, whose binding to LOX-1 is a well-established trigger for NADPH oxidase activation [63]. The ensuing stimulation of NOX isoforms, particularly NOX2 and NOX4, leads to sustained superoxide generation and downstream conversion into other ROS. Importantly, like as it happens in macrophages and endothelium, this mechanism can also be self-reinforcing, as oxidative stress promotes reciprocal upregulation of both LOX-1 and PCSK9, thereby amplifying the pro-oxidant loop [5].

In endothelial cells, PCSK9 has been shown to downregulate eNOS activity while upregulating NOX2 and NOX4, thereby reducing nitric oxide bioavailability and enhancing ROS generation [59]. In contrast, VSMC do not express eNOS under physiological conditions, and no direct evidence exists for PCSK9-driven modulation of NOX isoforms in this cell type, whose activity remains hypothetical.

A second proposed process, which remains to be experimentally validated, derives from the observation that the C-terminal domain of PCSK9 shares structural similarity with the adipokine resistin [64]. Resistin is known to activate oxidative signaling cascades in vascular cells, including MAPK and NF-κB pathways, leading to enhanced NADPH oxidase activity and ROS accumulation [65]. By analogy, PCSK9 may mimic this pro-oxidant role, potentially interacting with resistin-associated receptors or cofactors such as CAP1 to trigger comparable downstream redox pathways in VSMC, as discussed by Punch et al., 2022 [66].

Another plausible but untested mechanism in VSMC involves PCSK9-mediated impairment of mitochondrial quality control via sirtuin–autophagy dysregulation. In endothelial cells, PCSK9 inhibition boosts antioxidant defenses through **SIRT3** activity [67]. Separate studies establish that **SIRT4** protects VSMC from ox-LDL–induced oxidative stress [68]. It is therefore conceivable that PCSK9 may suppress SIRT4 expression in VSMC, thereby impairing mitophagy and facilitating ROS accumulation. However, no study to date has directly tested this PCSK9–SIRT4 axis, highlighting an important gap for future investigation.

Globally future research should aim to delineate the direct contribution of PCSK9 to NOX isoform activation and sirtuin-dependent mitochondrial quality control in VSMC, as current evidence remains largely inferential. Defining these pathways will be essential to clarify the role of PCSK9 in VSMC redox homeostasis.

### 5.3 Calcification

PCSK9 has recently been recognized as an active driver of vascular calcification, acting beyond lipid metabolism to influence VSMC fate, extracellular vesicle release, and inflammatory crosstalk [69]. Its actions span direct osteogenic reprogramming of arterial cells to broader paracrine and systemic effects, underscoring a multifaceted role in the initiation and propagation of arterial mineralization.

In human and rat VSMC, overexpression of intracellular PCSK9 represents the first direct demonstration of its pro-calcifying activity, as it enhances calcium deposition under high-phosphate conditions, induces osteogenic markers such as BMP2 and alkaline phosphatase, and concomitantly suppresses the anti-calcific proteins matrix Gla protein and osteopontin. These cells release an increased number of CD63/CD9/CD81-positive EV enriched in calcium and alkaline phosphatase, with PCSK9 itself incorporated into their cargo, whereas supplementation with extracellular recombinant PCSK9 alone fails to trigger mineralization, underscoring an intracellular and EV-mediated mechanism rather than a simple extracellular ligand effect [70].

EV have a uniquely altered molecular cargo (comprising 14 proteins and 6 microRNAs whose identity is still unknown) enriched for extracellular matrix structural constituents. Biologically, these EV induce endothelial cells to upregulate adhesion molecules, evoke greater inflammatory cytokine secretion and migratory behavior in monocytes, and enhance oxidized LDL uptake and inflammation in macrophages; they also promote macrophage recruitment in a zebrafish embryo model, establishing a pro-inflammatory circuitry that likely synergizes with mineral stress to facilitate calcification [47].

In addition to in vitro findings, in vivo evidence recently demonstrated that systemic delivery of an adeno-associated viral vector encoding the gain-of-function D377Y-pcsk9 murine mutant in wild-type markedly elevates circulating PCSK9 and induces progressive aortic calcification. Notably, this pathological response was significantly attenuated in sortilin-deficient animals, highlighting a potential PCSK9–sortilin axis that governs extracellular vesicle cargo loading during calcification [71]. Consistently, independent studies have established that sortilin directs the incorporation of tissue non-specific alkaline phosphatase into VSMC EV and is indispensable for their microcalcification capacity, thereby positioning sortilin as a potential bridge between PCSK9 signaling and vesicle-mediated mineral deposition [72].

PCSK9 has emerged as a central regulator of vascular calcification, exerting its influence through direct osteogenic reprogramming of vascular cells, modulation of EV biogenesis and cargo, and amplification of systemic lipid-inflammatory pathways. Future research should aim to comprehensively delineate the range of PCSK9's influence on calcifying activity and clarify whether its targeting can mitigate mineralization independently of cholesterol lowering.

### 5.4 PCSK9 feed-forward loops

**Fig. 4:**
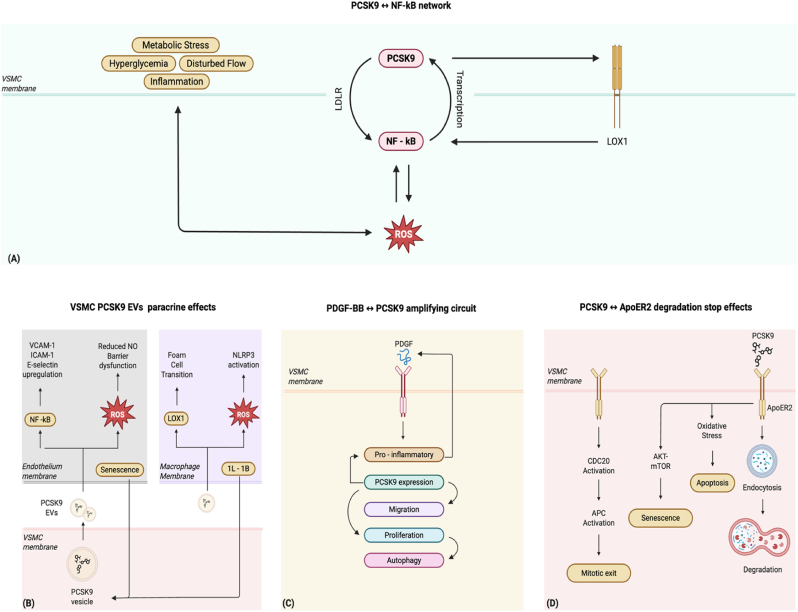
PCSK9 loops in VSMC. Note that NF – kB ROS interaction can be mediated by NLRP3 inflammasome.

PCSK9 reinforces feed-forward loops in VSMC, driving dedifferentiation, proliferation, migration, inflammation, lipid loading, and matrix remodeling that collectively accelerate atherogenesis (Fig. 4). Conversely, context-dependent inhibitory effects suggest that PCSK9 net impact reflects a balance between pro-synthetic activation and anti-proliferative restraint.

The PCSK9 ↔ NF-κB circle is the core from which expand self-enhancing ramifications that constitute a pathogenic molecular network. PCSK9 engages LDLR family receptors to activate NF-κB, which translocates to the nucleus where it promotes the transcription of pro-inflammatory cytokines, adhesion molecules, and PCSK9 itself [46] (Fig. 4A).

First, PCSK9 also promotes LOX-1 expression and increases ox-LDL uptake. Both events enhance NF-κB activity and stimulate ROS production. When flow is disturbed or inflammatory stimuli are present, ROS levels rise further and induce PCSK9 expression. This branch links hemodynamic stress to loss of redox balance and inflammatory activation of the vessel wall [14]. Second, PCSK9 interacts with the NLRP3 inflammasome. It promotes inflammasome priming and IL-1β release, which, in turn, increases PCSK9 expression. This bidirectional branch drives VSMC proliferation, migration, and inflammation, as shown in transplant vasculopathy models [29]. Third, metabolic stress provides another reinforcing ramification. Insulin resistance and hyperglycemia increase intracellular ROS, that activates PKC and MAPK cascades, which induce PCSK9 expression. Elevated PCSK9 sustains oxidative stress and prolongs PKC/MAPK activity [15]. Taken together, these interconnected loops establish PCSK9–NF-κB as the central hub of a self-reinforcing network that integrates hemodynamic stress, inflammasome signaling, and metabolic dysfunction into vascular inflammation and plaque progression.

A paracrine effect results from the interaction between extracellular vesicles loaded with PCSK9 (EV-PCSK9) produced by VSMC and vascular inflammation (Fig. 4B). EV-PCSK9 from VSMC amplify inflammatory activation in endothelial cells and monocytes and enhance ox-LDL uptake in macrophages. In turn, the inflammatory environment induces PCSK9 expression in VSMC. This two-way interaction establishes a self-perpetuating pathway within atherosclerotic plaques, maintaining both inflammation and upregulation of PCSK9 [47].

The PCSK9 ↔ growth factor (PDGF-BB) loop establishes a mutual reinforcement that consolidates the synthetic phenotype of VSMC (Fig. 4C). In this amplifying circuit, PDGF-BB upregulates PCSK9 expression in VSMC, and PCSK9, in turn, enhances proliferation, migration, and autophagy independently of LDL-C. Although this is not a fully closed feedback loop, since PCSK9 does not directly upregulate PDGF-BB expression but rather amplifies its downstream effects, it highlights the cooperative interaction between growth-factor signaling and PCSK9 in driving VSMC activation [27].

Instead, **PCSK9 ↔ ApoER2 degradation stop effects** sees PCSK9 promoting the degradation of ApoER2 in VSMC, thereby disrupting receptor signaling and triggering aberrant DNA replication without cytokinesis, leading to polyploidization, senescence, and ultimately apoptosis (Fig. 4D). Biologically, this negative feedback may limit lesion cellularity but simultaneously it promotes the accumulation of senescent, dysfunctional VSMC that compromise plaque stability [35].

These pathways underscore the pathophysiological significance of PCSK9 as a central amplifier of VSMC dysfunction, integrating hemodynamic, metabolic, and inflammatory cues into self-reinforcing processes that drive lesion growth and destabilization, while also exerting context-dependent inhibitory effects through senescence and apoptosis. Future research should aim to dissect the temporal and spatial balance of these opposing influences within the atherosclerotic plaque.

## 6 PCSK9 inhibition to fight atherosclerosis

Beyond its canonical role in LDL receptor regulation, PCSK9 directly promotes atherogenesis by amplifying vascular inflammation and disturbing VSMC homeostasis. Pharmacological strategies that suppress PCSK9 expression or intercept its signaling show differential potential: approaches targeting upstream synthesis or alternative binding partners appear most comprehensive, whereas ligand-neutralizing modalities may confer only partial protection against VSMC-mediated plaque progression [73,74]. For instance, recent multi-level evidence indicates that PCSK9 inhibitors ameliorate arterial stiffness in patients with acute coronary syndrome (ACS). Mendelian randomization, clinical cohort data, and in vitro studies collectively show that PCSK9 inhibition leads to improved pulse wave velocity and overall arterial compliance [75].

As the first class of agents under consideration, fully human monoclonal antibodies such as evolocumab and alirocumab act by binding PCSK9 and preventing its interaction with downstream targets. These antibodies primarily target circulating PCSK9, the soluble form secreted predominantly by the liver. Given their limited tissue permeability, these agents are unlikely to neutralize intracellular or locally expressed PCSK9 within the vascular wall. Nevertheless, by markedly reducing systemic PCSK9 levels, they diminish paracrine exposure of vascular cells to PCSK9 and thereby attenuate downstream inflammatory signaling [76,77]. Although originally designed to block PCSK9–LDLR binding and thereby restore receptor recycling with marked reductions in LDL-C, their sequestration of PCSK9 also limits its non-LDLR interactions within the vascular wall [78]. These antibodies attenuate pathogenic signaling, as in atherosclerotic mice where the treatment halved plaque macrophage burden and decreased pro-inflammatory chemokine secretion (CXCL1, CXCL3, CXCL10) from ECs and VSMC. Consistently, PCSK9 deficiency has been associated with diminished adhesion molecule expression, with *Pcsk9*^−/−^ mice exhibiting significantly lower VCAM-1 levels in the aortic wall [79]. In carotid ligation models, both PCSK9 deficiency and evolocumab treatment reduced neointimal hyperplasia, accompanied by lower proliferative markers, suppressed autophagy, and diminished VSMC migration in vitro [27]. Also apoptosis remains inhibited, thereby supporting the maintenance of plaque-stabilizing VSMC in advanced lesions [38]. Clinically, imaging studies have demonstrated that adding evolocumab or alirocumab to statin therapy increases fibrous cap thickness and enhances plaque stability, with benefits that surpass those attributable to LDL-C lowering alone [80]. Alirocumab induced improvements in plaque morphology were also attested in the PACMAN-ARI clinical trial [81]. While therapeutic effects on systemic inflammation are modest, smaller mechanistic investigations suggest local reductions in cytokine activity and plaque inflammatory signaling [82]. Improvements in vascular function have also been reported, with evidence linking PCSK9 inhibition to reduced arterial stiffness [83]. Collectively, these findings indicate that monoclonal antibodies not only achieve potent LDL-C reduction but also confer direct vessel-wall benefits, attenuating inflammation and supporting plaque stability through preservation of VSMC integrity.

Unlike monoclonal antibodies, which neutralize circulating PCSK9, other approaches act within the cell, reducing intracellular PCSK9 synthesis and accumulation in tissues such as VSMC and hepatocytes. Selective inhibition of hepatic PCSK9 profoundly lowers circulating LDL cholesterol and reduces atherosclerotic plaque burden [84]. Beyond its hepatic role, PCSK9 is also expressed in vascular cells, where it contributes to local inflammatory signaling, lipid uptake, and plaque instability independently of blood lipid levels. Consequently, systemic lowering of PCSK9 levels not only improves lipid metabolism but also hinders vascular inflammation and promotes a more stable plaque phenotype. Nonetheless, residual PCSK9 activity within vascular tissue may continue to modulate macrophage recruitment, oxidative stress, and extracellular matrix remodeling [46]. A molecular example of this involves the use of PCSK9-targeted autophagosome-tethering compounds, designed to direct PCSK9 toward lysosomal degradation via the autophagy pathway. These molecules reduce intracellular and circulating PCSK9, thereby increasing LDL receptor expression and promoting LDL clearance. In experimental models, autophagy tethering compounds (ATTEC) improved lipid profiles, diminished atherosclerotic plaque formation, and reduced vascular inflammation, represent a promising strategy [85].

Liraglutide, a GLP-1 receptor agonist, has recently emerged for protecting VSMC from the harmful effects of homocysteine, a key risk factor for atherosclerosis. Homocysteine exposure induces proliferation, migration, phenotypic switching, and ultimately plaque development. Liraglutide counteracted these changes by suppressing intracellular PCSK9 expression and restoring LDL receptor levels within VSMC, thereby reducing lipid imbalance and inflammatory signaling. These findings suggest that liraglutide exerts direct vascular benefits beyond its metabolic actions, pointing to modulation of the PCSK9/LDLR pathway as a novel protective mechanism [86].

RNA-based strategies are also being developed to silence hepatic PCSK9 expression and achieve sustained suppression of circulating PCSK9 protein levels. Inclisiran, a GalNAc-conjugated small interfering RNA administered by subcutaneous injection, directs the degradation of PCSK9 mRNA in hepatocytes, producing reductions in plasma PCSK9 of more than 80 % and lowering LDL-C by approximately 50 % [87]. Antisense oligonucleotides targeting PCSK9 (e.g., AZD1222/ION449) are currently under clinical experimentation with promising expectations [88]. Concerning pre-clinical results, hepatic silencing of PCSK9 through adeno-associated virus–mediated shRNA delivery in hyperlipidemic mice produces not only reductions in circulating LDL but also attenuation of atherosclerotic burden, macrophage infiltration, and vascular inflammation. These vascular improvements likely result from diminished systemic PCSK9 exposure rather than direct effects on VSMC-derived PCSK9 [89]. Clinically, inclisiran achieves substantial LDL-C reductions and is now approved for use in patients at high cardiovascular risk. By providing sustained suppression of PCSK9, in contrast to the fluctuating pharmacodynamic profile of monoclonal antibodies, inclisiran achieves a more stable modulation of PCSK9 levels [90]. Several ongoing trials are specifically assessing vascular endpoints such as carotid plaque burden and composition [91]. Overall, RNA-based therapies that silence PCSK9 provide a broad means to block both lipid-dependent and independent effects, attenuating VSMC degeneration and supporting plaque stabilization, characterized by reinforced fibrous caps and reduced inflammatory infiltration.

Genome editing has emerged as a potential one-time approach for permanent hepatic PCSK9 inactivation. In vivo application of CRISPR/Cas9 or base-editing platforms can introduce loss-of-function variants in the PCSK9 gene in hepatocytes, offering a durable alternative to siRNA or antisense oligonucleotides, which necessitate repeated administration [92]. Beyond sustained LDL-C reduction, animal studies consistently demonstrate attenuated atherosclerosis and a more stable plaque phenotype, with fewer necrotic cores and increased collagen, due to long-term suppression of systemic PCSK9 activity [93]. Safety concerns remain, particularly regarding potential off-target edits, liver enzyme elevations, and immune responses. Nevertheless, advances in delivery and editing precision have encouraged early clinical translation, with Verve Therapeutics initiating first-in-human trials of in vivo base editing to permanently silence hepatic PCSK9 [94].

One innovative approach is the use of peptide mimetics derived from the LDLR EGF-A loop, which bind circulating PCSK9 and block its interaction with native receptors. Early examples such as Pep2-8 inhibited nearly 90 % of PCSK9–LDLR binding in vitro, without affecting directly its vascular actions [95]. The CAP1-Fc decoy receptor represents a more selective biologic strategy. This fusion protein, comprising the extracellular PCSK9-binding domain of CAP1 linked to an IgG Fc fragment, sequesters extracellular PCSK9 and prevents its interaction with cellular CAP1. In doing so, it effectively blocks PCSK9-driven inflammatory signaling (more than evolocumab). Moreover, given CAP1's role in PCSK9-mediated LDLR internalization, CAP1-Fc is capable of inhibiting both the pro-inflammatory and receptor-degradative functions of PCSK9 [46]. Therapeutic vaccination against circulating PCSK9 represents another peptide-based strategy, exemplified by AT04A, which couples a short PCSK9-derived peptide to a carrier immunogen to elicit endogenous antibody production. In hyperlipidemic mouse models, such vaccination induced persistent anti-PCSK9 titers, lowered LDL-C by more than 50 %, and significantly attenuated atherosclerosis [96]. Beyond lipid lowering, vaccinated animals displayed reduced arterial ICAM-1 expression and diminished monocyte adhesion, as well as decreased plaque NLRP3 inflammasome activity and IL-1β expression, highlighting PCSK9's immunomodulatory role in promoting endothelial activation and innate immune signaling [96]. By neutralizing PCSK9 through polyclonal antibodies, vaccination is expected to confer benefits comparable to monoclonal antibodies, also including VSMC protection.

As a final therapeutic frontier, orally administered small-molecule PCSK9 inhibitors offer a compelling alternative to injectable biologics. Agents such as AZD0780, which competitively binds PCSK9's C-terminal domain, and 7030B-C5, which suppresses hepatic PCSK9 transcription through FoxO1/HNF1α modulation, not only lower LDL-C but also mitigate vascular inflammation and VSMC activation in preclinical models due to low systemic PCSK9 levels [97]. Natural compounds like berberine similarly downregulate PCSK9 expression, although less potently. Lastly, Enlicitide (MK-0616) is a next-generation, orally bioavailable macrocyclic peptide that inhibits circulating PCSK9, preventing its interaction with the LDL receptor and thereby enhancing LDL-C clearance. Formulated with absorption enhancers to ensure effective intestinal uptake, it has achieved LDL-C reductions of up to 60–65 % in clinical trials with an excellent safety and tolerability profile. Currently in phase III evaluation, Enlicitide represents the most advanced oral PCSK9 inhibitor to date, offering a convenient and effective alternative to injectable biologics and holding promise for improved treatment adherence and accessibility in lipid-lowering therapy [98]. If validated in humans, these oral therapies could integrate lipid reduction with direct vascular protection [98]. Current therapeutic strategies against PCSK9 have already proven effective in lowering LDL cholesterol and, increasingly, in mitigating vascular inflammation, VSMC dysfunction, and plaque instability. Future research should prioritize approaches that target PCSK9's non-LDLR signaling, particularly its inflammatory and VSMC-modulatory pathways, while advancing novel modalities such as RNA-based therapies, gene editing, and oral small-molecule inhibitors toward safe, durable, and accessible clinical translation.

## 7 Conclusions

This project aimed to review the role of PCSK9 in medicine. Beyond its canonical role as a liver regulator of LDL-R degradation, PCSK9 is increasingly recognized as a central actor in VSMC biology. Within the arterial wall, VSMC constitute the predominant extrahepatic source of PCSK9, where its actions extend beyond lipid handling to encompass autocrine and paracrine regulation of cell fate. By engaging signaling cascades such as PI3K–AKT–mTOR, activating the NLRP3 inflammasome, upregulating scavenger receptors, and facilitating extracellular vesicle release, PCSK9 profoundly disrupts VSMC homeostasis. These processes collectively drive the transition of VSMC from a contractile to synthetic, inflammatory, and osteogenic phenotypes, promoting vascular remodeling and destabilization. Furthermore, PCSK9-mediated reprogramming of VSMC enhances pathological communication with endothelial and immune compartments, positioning PCSK9 as a critical integrator of vascular dysfunction and atherogenic progression.

By fostering foam cell formation, amplifying oxidative stress, sustaining vascular inflammation, and driving calcific remodeling, PCSK9 accelerates plaque expansion while compromising structural stability. These pathological processes contribute not only to lesion growth but also to fibrous cap thinning and heightened vulnerability to rupture [99]. Therapeutically, inhibition of PCSK9 has been shown to mitigate these adverse effects in addition to lowering circulating LDL-C. Monoclonal antibodies such as evolocumab and alirocumab have demonstrated the capacity to increase fibrous cap thickness and attenuate inflammatory activity, while RNA-based agents including inclisiran provide durable suppression of PCSK9 with consistent vascular benefit. Complementary preclinical strategies, ranging from gene-editing platforms to peptide vaccines and CAP1-targeted biologics, further highlight the therapeutic potential of directly intercepting PCSK9-driven vascular pathology. Collectively, these interventions define PCSK9 blockade as a dual-action approach, simultaneously reducing systemic cholesterol burden and directly protecting the arterial wall.

Despite these advances, significant knowledge gaps remain. Future research must delineate the relative weight of PCSK9's lipid-dependent versus direct vascular actions, with emphasis on human VSMC and in vivo tissues. Dissecting intracellular signaling networks, extracellular vesicle cargo composition, and the interplay with metabolic stressors will be essential to define context-dependent outcomes. In addition, refining strategies that selectively block non-LDLR pathways (particularly inflammatory, pro-oxidant, and pro-calcific circuits) could yield targeted therapies that stabilize plaques independently of cholesterol lowering. Precision approaches, integrating spatial and temporal resolution of PCSK9 activity within vascular niches, alongside advanced RNA and genome-editing modalities, will be central to the next stage of translation.

Ultimately, the therapeutic horizon lies in shifting PCSK9 biology from a systemic cholesterol target toward a multifaceted vascular modulator, with the goal of attenuating atherosclerosis at its genetical roots.

## Declaration of competing interest

The authors declare that they have no known competing financial interests or personal relationships that could have appeared to influence the work reported in this paper.

## References

[1] Stewart J., Manmathan G., Wilkinson P. (2017). Primary prevention of cardiovascular disease: a review of contemporary guidance and literature. JRSM Cardiovasc Dis.

[2] Ajoolabady A., Pratico D., Lin L., Mantzoros C.S., Bahijri S., Tuomilehto J. (2024 Nov 11). Inflammation in atherosclerosis: pathophysiology and mechanisms. Cell Death Dis.

[3] Seidah N.G., Prat A. (2022 May 12). The multifaceted biology of PCSK9. Endocr Rev.

[4] Dong B., Wu M., Li H., Kraemer F.B., Adeli K., Seidah N.G. (2010 June). Strong induction of PCSK9 gene expression through HNF1alpha and SREBP2: mechanism for the resistance to LDL-cholesterol lowering effect of statins in dyslipidemic hamsters. J Lipid Res.

[5] Ding Z., Liu S., Wang X., Deng X., Fan Y., Shahanawaz J. (2015 Sept 1). Cross-talk between LOX-1 and PCSK9 in vascular tissues. Cardiovasc Res.

[6] Ridker P.M., Revkin J., Amarenco P., Brunell R., Curto M., Civeira F. (2017 Apr 20). Cardiovascular efficacy and safety of bococizumab in high-risk patients. N Engl J Med.

[7] Sabatine M.S., Giugliano R.P., Keech A.C., Honarpour N., Wiviott S.D., Murphy S.A. (2017 May 4). Evolocumab and clinical outcomes in patients with cardiovascular disease. N Engl J Med.

[8] Schwartz G.G., Szarek M., Bhatt D.L., Bittner V.A., Bujas-Bobanovic M., Diaz R. (2023 Mar 5). Transiently achieved very low LDL-cholesterol levels by statin and alirocumab after acute coronary syndrome are associated with cardiovascular risk reduction: the ODYSSEY OUTCOMES trial. Eur Heart J.

[9] Kong P., Cui Z.Y., Huang X.F., Zhang D.D., Guo R.J., Han M. (2022 Apr 22). Inflammation and atherosclerosis: signaling pathways and therapeutic intervention. Sig Transduct Target Ther.

[10] Iida Y., Tanaka H., Sano H., Suzuki Y., Shimizu H., Urano T. (2018 Apr). Ectopic expression of PCSK9 by smooth muscle cells contributes to aortic dissection. Ann Vasc Surg.

[11] Wirka R.C., Wagh D., Paik D.T., Pjanic M., Nguyen T., Miller C.L. (2019 Aug). Atheroprotective roles of smooth muscle cell phenotypic modulation and the TCF21 disease gene as revealed by single-cell analysis. Nat Med.

[12] Ferri N., Tibolla G., Pirillo A., Cipollone F., Mezzetti A., Pacia S. (2012 Feb). Proprotein convertase subtilisin kexin type 9 (PCSK9) secreted by cultured smooth muscle cells reduces macrophages LDLR levels. Atherosclerosis.

[13] Zeng J., Tao J., Xi L., Wang Z., Liu L. (2021 Apr). PCSK9 mediates the oxidative low-density lipoprotein-induced pyroptosis of vascular endothelial cells via the UQCRC1/ROS pathway. Int J Mol Med.

[14] Ding Z., Liu S., Wang X., Deng X., Fan Y., Sun C. (2015 Mar 20). Hemodynamic shear stress via ROS modulates PCSK9 expression in human vascular endothelial and smooth muscle cells and along the mouse aorta. Antioxid Redox Signal.

[15] Barale C., Tempesta G., Melchionda E., Morotti A., Frascaroli C., Danzero A.C. (2025 Jan). PCSK9 expression in vascular smooth muscle cells: role of insulin resistance and high glucose. Int J Mol Sci.

[16] Melchionda E., Barale C., Tempesta G., Sornatale M., Russo I. (2023 Aug 1). In aortic vascular smooth muscle cells high glucose increases PCSK9 expression: role of PCSK9 inhibitors. Atherosclerosis.

[17] Ricci C., Ruscica M., Camera M., Rossetti L., Macchi C., Colciago A. (2018 Feb 2). PCSK9 induces a pro-inflammatory response in macrophages. Sci Rep.

[18] Jaén R.I., Povo-Retana A., Rosales-Mendoza C., Capillas-Herrero P., Sánchez-García S., Martín-Sanz P. (2022 Jan). Functional crosstalk between PCSK9 internalization and pro-inflammatory activation in human macrophages: role of reactive oxygen species release. Int J Mol Sci.

[19] Seidah N.G., Garçon D. (2022). Expanding biology of PCSK9: roles in atherosclerosis and beyond. Curr Atheroscler Rep.

[20] Liu A., Frostegård J. (2018 Apr). PCSK9 plays a novel immunological role in oxidized LDL-Induced dendritic cell maturation and activation of T cells from human blood and atherosclerotic plaque. J Intern Med.

[21] Tan D., Yang X., Yang J., Fan G., Xiong G. (2025 Feb). PCSK9 in vascular aging and age-related diseases. Aging Dis.

[22] Puteri M.U., Azmi N.U., Ridwan S., Iqbal M., Fatimah T., Rini T.D.P. (2022 Aug 10). Recent update on PCSK9 and Platelet activation experimental research methods: in vitro and in vivo studies. J Cardiovasc Dev Dis.

[23] Di Costanzo A., Indolfi C., Sorrentino S., Esposito G., Spaccarotella C.A.M. (2023 July 21). The effects of statins, ezetimibe, PCSK9-Inhibitors, inclisiran, and icosapent ethyl on platelet function. Int J Mol Sci.

[24] Petersen-Uribe Á., Kremser M., Rohlfing A.K., Castor T., Kolb K., Dicenta V. (2021 Jan). Platelet-derived PCSK9 is associated with LDL metabolism and modulates atherothrombotic mechanisms in coronary artery disease. Int J Mol Sci.

[25] Sundararaman S.S., Peters L.J.F., Nazir S., Marquez A.B., Bouma J.E., Bayasgalan S. (2021 Dec 1). PCSK9 imperceptibly affects chemokine receptor expression in vitro and in vivo. Int J Mol Sci.

[26] Ferri N., Marchianò S., Tibolla G., Baetta R., Dhyani A., Ruscica M. (2016 Oct). PCSK9 knock-out mice are protected from neointimal formation in response to perivascular carotid collar placement. Atherosclerosis.

[27] Zhang Q., Miao M., Cao S., Liu D., Cao Z., Bai X. (2025 Jan). PCSK9 promotes vascular neointimal hyperplasia through non-lipid regulation of vascular smooth muscle cell proliferation, migration, and autophagy. Biochem Biophys Res Commun.

[28] Nelson P.R., Yamamura S., Kent K.C. (1997 July). Platelet-derived growth factor and extracellular matrix proteins provide a synergistic stimulus for human vascular smooth muscle cell migration. J Vasc Surg.

[29] Zou Y., Chen Z., Zhang X., Yu J., Xu H., Cui J. (2022). Targeting PCSK9 ameliorates graft vascular disease in mice by inhibiting NLRP3 inflammasome activation in vascular smooth muscle cells. Front Immunol.

[30] Bond M., Chase A.J., Baker A.H., Newby A.C. (2001 June). Inhibition of transcription factor NF-kappaB reduces matrix metalloproteinase-1, -3 and -9 production by vascular smooth muscle cells. Cardiovasc Res.

[31] Huang L., Li Y., Cheng Z., Lv Z., Luo S., Xia Y. (2023 Feb). PCSK9 promotes endothelial dysfunction during sepsis via the TLR4/MyD88/NF-κB and NLRP3 pathways. Inflammation.

[32] Scalise V., Sanguinetti C., Neri T., Cianchetti S., Lai M., Carnicelli V. (2021 Jan). PCSK9 induces tissue factor expression by activation of TLR4/NFkB signaling. Int J Mol Sci.

[33] Liu Y., Zhao Y., Feng P., Jiang H. (2023 July). PCSK9 inhibitor attenuates atherosclerosis by regulating SNHG16/EZH2/TRAF5-mediated VSMC proliferation, migration, and foam cell formation. Cell Biol Int.

[34] Allard D., Figg N., Bennett M.R., Littlewood T.D. (2008 July 11). Akt regulates the survival of vascular smooth muscle cells via inhibition of FoxO3a and GSK3. J Biol Chem.

[35] Guo Y., Tang Z., Yan B., Yin H., Tai S., Peng J. (2022 Jan). PCSK9 (proprotein convertase subtilisin/kexin type 9) triggers vascular smooth muscle cell senescence and apoptosis: implication of its direct role in degenerative vascular disease. Arterioscler Thromb Vasc Biol.

[36] Komaravolu R.K., Waltmann M.D., Konaniah E., Jaeschke A., Hui D.Y. (2019 Oct). ApoER2 (apolipoprotein E Receptor-2) deficiency accelerates smooth muscle cell senescence via cytokinesis impairment and promotes fibrotic neointima after vascular injury. Arterioscler Thromb Vasc Biol.

[37] Kaistha A., Oc S., Garrido A.M., Taylor J.C.K., Imaz M., Worssam M.D. (2025 Aug 1). Premature cell senescence promotes vascular smooth muscle cell phenotypic modulation and resistance to re-differentiation. Cardiovasc Res.

[38] Xu R., Li T., Luo J., Zhang X., Wang T., Wang Y. (2024 Feb 25). PCSK9 increases vulnerability of carotid plaque by promoting mitochondrial dysfunction and apoptosis of vascular smooth muscle cells. CNS Neurosci Ther.

[39] Ding Z., Liu S., Wang X., Mathur P., Dai Y., Theus S. (2016 Dec 20). Cross-talk between PCSK9 and damaged mtDNA in vascular smooth muscle cells: role in apoptosis. Antioxid Redox Signal.

[40] Pang H., Fu B., Wang P., Meng Y., Xie P., Hu X. (2025 Oct). WTAP silencing protects human aortic smooth muscle cells from angiotensin II-induced senescence, apoptosis, ferroptosis, and inflammation by regulating PCSK9. J Bioenerg Biomembr.

[41] Greaves D.R., Gordon S. (2005 Jan). Thematic review series: the immune system and atherogenesis. Recent insights into the biology of macrophage scavenger receptors. J Lipid Res.

[42] Tam J., Thankam F., Agrawal D.K., Radwan M.M. (2021 Oct). Critical role of LOX-1-PCSK9 axis in the pathogenesis of atheroma formation and its instability. Heart Lung Circ.

[43] Demers A., Samami S., Lauzier B., Des Rosiers C., Ngo Sock E.T., Ong H. (2015 Dec). PCSK9 induces CD36 degradation and affects long-chain fatty acid uptake and triglyceride metabolism in adipocytes and in mouse liver. Arterioscler Thromb Vasc Biol.

[44] Ding Z., Liu S., Wang X., Theus S., Deng X., Fan Y. (2018 July 1). PCSK9 regulates expression of scavenger receptors and ox-LDL uptake in macrophages. Cardiovasc Res.

[45] Adorni M.P., Cipollari E., Favari E., Zanotti I., Zimetti F., Corsini A. (2017 Jan). Inhibitory effect of PCSK9 on Abca1 protein expression and cholesterol efflux in macrophages. Atherosclerosis.

[46] Shin D., Kim S., Lee H., Lee H.C., Lee J., Park H woo (2024 Mar 30). PCSK9 stimulates syk, PKCδ, and NF-κB, leading to atherosclerosis progression independently of LDL receptor. Nat Commun.

[47] Greco M.F., Rizzuto A.S., Zarà M., Cafora M., Favero C., Solazzo G. (2022 Oct 28). PCSK9 confers inflammatory properties to extracellular vesicles released by vascular smooth muscle cells. Int J Mol Sci.

[48] Lupo M.G., Marchianò S., Adorni M.P., Zimetti F., Ruscica M., Greco M.F. (2021 Jan). PCSK9 induces rat smooth muscle cell proliferation and counteracts the pleiotropic effects of simvastatin. Int J Mol Sci.

[49] Ding Z., Pothineni N.V.K., Goel A., Lüscher T.F., Mehta J.L. (2020 Apr 1). PCSK9 and inflammation: role of shear stress, pro-inflammatory cytokines, and LOX-1. Cardiovasc Res.

[50] Xiang Q., Liu W.F., Zeng J.L., Deng Y.M., Peng J., Liu H.T. (2021). Effect of PCSK9 on vascular smooth muscle cell functions: a new player in atherosclerosis. Curr Med Chem.

[51] Hoogeveen R.M., Opstal T.S.J., Kaiser Y., Stiekema L.C.A., Kroon J., Knol R.J.J. (2019 Dec). PCSK9 antibody alirocumab attenuates arterial Wall inflammation without changes in circulating inflammatory markers. JACC Cardiovasc Imaging.

[52] Lee J.H., Shores K.L., Breithaupt J.J., Lee C.S., Fodera D.M., Kwon J.B. (2023 Dec). PCSK9 activation promotes early atherosclerosis in a vascular microphysiological system. APL Bioeng.

[53] Puspitasari Y.M., Ministrini S., Liberale L., Vukolic A., Baumann-Zumstein P., Holy E.W. (2023 Dec). Antibody-mediated PCSK9 neutralization worsens outcome after bare-metal stent implantation in mice. Vascul Pharmacol.

[54] Choi J.Y., Gwon J.G. (2025 Sept 11). Genetic pathogenesis of abdominal aortic aneurysm and the role of PCSK9 (Proprotein convertase Subtilisin/Kexin type 9). Vasc Specialist Int.

[55] Ragusa R., Basta G., Neglia D., De Caterina R., Del Turco S., Caselli C. (2021 Apr). PCSK9 and atherosclerosis: looking beyond LDL regulation. Eur J Clin Invest.

[56] Yurtseven E., Ural D., Baysal K., Tokgözoğlu L. (2020 Sept 1). An update on the role of PCSK9 in atherosclerosis. J Atheroscler Thromb.

[57] Cariou B., Si-Tayeb K., Le May C. (2015 June). Role of PCSK9 beyond liver involvement. Curr Opin Lipidol.

[58] Wang Y., Cao S., Wang Z., Li C., Ye J., Liu Y. (2025 Mar 1). PCSK9 affects vascular senescence through the SIRT1 pathway. Exp Gerontol.

[59] Liu S., Wu J., Stolarz A., Zhang H., Boerma M., Byrum S.D. (2023). PCSK9 attenuates efferocytosis in endothelial cells and promotes vascular aging. Theranostics.

[60] Grune J., Meyborg H., Bezhaeva T., Kappert K., Hillmeister P., Kintscher U. (2017 Apr 1). PCSK9 regulates the chemokine receptor CCR2 on monocytes. Biochem Biophys Res Commun.

[61] Li Z., Zhu L., Xu Y., Zhang Y., Liu Y., Sun H. (2024 Dec). Pleiotropic effects of PCSK9 inhibitors on cardio-cerebrovascular diseases. Biomedicines.

[62] Batty M., Bennett M.R., Yu E. (2022 Jan). The role of oxidative stress in atherosclerosis. Cells.

[63] Pirillo A., Norata G.D., Catapano A.L. (2013). LOX-1, OxLDL, and atherosclerosis. Mediators Inflamm.

[64] Hampton E.N., Knuth M.W., Li J., Harris J.L., Lesley S.A., Spraggon G. (2007 Sept 11). The self-inhibited structure of full-length PCSK9 at 1.9 Å reveals structural homology with resistin within the C-terminal domain. Proc Natl Acad Sci U S A.

[65] Wang B.W., Chang H., Shyu K.-G. (2009 Oct 26). Regulation of resistin by cyclic mechanical stretch in cultured rat vascular smooth muscle cells. Clin Sci (Lond).

[66] Punch E., Klein J., Diaba-Nuhoho P., Morawietz H., Garelnabi M. (2022 Feb). Effects of PCSK9 targeting: alleviating oxidation, inflammation, and atherosclerosis. J Am Heart Assoc.

[67] D'Onofrio N., Prattichizzo F., Marfella R., Sardu C., Martino E., Scisciola L. (2023). SIRT3 mediates the effects of PCSK9 inhibitors on inflammation, autophagy, and oxidative stress in endothelial cells. Theranostics.

[68] Hao Y., Li W. (2024). Regulatory factor X7 represses Ox-LDL-Induced proliferation and migration of VSMCs via SIRT4-Mediated inactivation of JAK2/STAT3 pathway. Int Heart J.

[69] Poggio P., Songia P., Cavallotti L., Barbieri S.S., Zanotti I., Arsenault B.J. (2018 Dec 18). PCSK9 involvement in aortic valve calcification. J Am Coll Cardiol.

[70] Lupo M.G., Bressan A., Donato M., Canzano P., Camera M., Poggio P. (2022 Apr). PCSK9 promotes arterial medial calcification. Atherosclerosis.

[71] Goettsch C., Hutcheson J.D., Hagita S., Rogers M.A., Creager M.D., Pham T. (2016 Aug 1). A single injection of gain-of-function mutant PCSK9 adeno-associated virus vector induces cardiovascular calcification in mice with no genetic modification. Atherosclerosis.

[72] Goettsch C., Hutcheson J.D., Aikawa M., Iwata H., Pham T., Nykjaer A. (2016 Apr 1). Sortilin mediates vascular calcification via its recruitment into extracellular vesicles. J Clin Investig.

[73] Liberale L., Montecucco F., Camici G.G., Dallegri F., Vecchie A., Carbone F. (2017). Treatment with proprotein Convertase Subtilisin/kexin type 9 (PCSK9) inhibitors to reduce cardiovascular inflammation and outcomes. Curr Med Chem.

[74] Ogura M. (2018 Jan). PCSK9 inhibition in the management of familial hypercholesterolemia. J Cardiol.

[75] Xu L., Wang L., Wang Y., Wang Y., Jiang Y., Du P. (2024). PCSK9 inhibitors ameliorate arterial stiffness in ACS patients: evidences from Mendelian randomization, a retrospective study and basic experiments. Front Med.

[76] Keating G.M. (2016 Feb). Evolocumab: a review in hyperlipidemia. Am J Cardiovasc Drugs.

[77] Tomlinson B., Hu M., Zhang Y., Chan P., Liu Z.M. (2017 May). Alirocumab for the treatment of hypercholesterolemia. Expert Opin Biol Ther.

[78] Leucker T.M., Gerstenblith G., Schär M., Brown T.T., Jones S.R., Afework Y. (2020 July 21). Evolocumab, a PCSK9‐Monoclonal antibody, rapidly reverses coronary artery endothelial dysfunction in people living with HIV and people with dyslipidemia. J Am Heart Assoc.

[79] Schuster S., Rubil S., Endres M., Princen H.M.G., Boeckel J.N., Winter K. (2019 July 31). Anti-PCSK9 antibodies inhibit pro-atherogenic mechanisms in APOE∗3Leiden.CETP mice. Sci Rep.

[80] Nicholls S.J., Kataoka Y., Nissen S.E., Prati F., Windecker S., Puri R. (2022 July). Effect of evolocumab on coronary plaque phenotype and burden in statin-treated patients following myocardial infarction. JACC Cardiovasc Imaging.

[81] Räber L., Ueki Y., Otsuka T., Losdat S., Häner J.D., Lonborg J. (2022 May 10). Effect of Alirocumab added to high-intensity Statin therapy on coronary atherosclerosis in patients with acute myocardial infarction: the PACMAN-AMI randomized clinical trial. JAMA.

[82] Cao Y.X., Li S., Liu H.H., Li J.J. (2018 Oct 4). Impact of PCSK9 monoclonal antibodies on circulating hs-CRP levels: a systematic review and meta-analysis of randomised controlled trials. BMJ Open.

[83] Silla A., Fogacci F., Punzo A., Hrelia S., Simoni P., Caliceti C. (2023 Feb 25). Treatment with PCSK9 inhibitor evolocumab improves vascular oxidative stress and arterial stiffness in hypercholesterolemic patients with high cardiovascular risk. Antioxidants.

[84] Wang X., Chen X., Zhang X., Su C., Yang M., He W. (2020 Feb 12). A small-molecule inhibitor of PCSK9 transcription ameliorates atherosclerosis through the modulation of FoxO1/3 and HNF1α. EBioMedicine.

[85] Wu H., Zhang Z., Xue Y., Guo J., Ouyang Z., Cao Z. (2025 Apr 24). PCSK9 targeted autophagosome-tethering compounds: design, synthesis, and antiatherosclerosis evaluation. J Med Chem.

[86] ∗Ji J, Feng M, Niu X, Zhang X, Wang Y. Liraglutide blocks the proliferation, migration and phenotypic switching of Homocysteine (Hcy)-induced vascular smooth muscle cells (VSMCs) by suppressing proprotein convertase subtilisin kexin9 (PCSK9)/low-density lipoprotein receptor (LDLR). Bioengineered. 12(1):8057–8066. 10.1080/21655979.2021.1982304 PMC8806487 34666623

[87] Nishikido T. (2023 Jan 30). Clinical potential of inclisiran for patients with a high risk of atherosclerotic cardiovascular disease. Cardiovasc Diabetol.

[88] Mohamed F., Mansfield B., Raal F.J. (2023 Aug 2). Targeting PCSK9 and beyond for the management of low-density lipoprotein cholesterol. J Clin Med.

[89] Chen X., Sun M., Ma X., Ma Y., Chen B. (2025 Mar 13). Silencing hepatic PCSK9 via novel chimeric AAV8 mitigates the progression of atherosclerosis by inhibiting inflammation in ApoE−/− mice. Mol Ther, Methods Clin Dev.

[90] Gosselin N.H., Schuck V.J.A., Barriere O., Kulmatycki K., Margolskee A., Smith P. (2023 Feb). Translational population-pharmacodynamic modeling of a novel long-acting siRNA therapy, inclisiran, for the treatment of hypercholesterolemia. Clin Pharmacol Ther.

[91] Ray K.K., Troquay R.P.T., Visseren F.L.J., Leiter L.A., Scott Wright R., Vikarunnessa S. (2023 Feb). Long-term efficacy and safety of inclisiran in patients with high cardiovascular risk and elevated LDL cholesterol (ORION-3): results from the 4-year open-label extension of the ORION-1 trial. Lancet Diabetes Endocrinol.

[92] Musunuru K., Chadwick A.C., Mizoguchi T., Garcia S.P., DeNizio J.E., Reiss C.W. (2021 May). In vivo CRISPR base editing of PCSK9 durably lowers cholesterol in Primates. Nature.

[93] Ding Q., Strong A., Patel K.M., Ng S.L., Gosis B.S., Regan S.N. (2014 Aug 15). Permanent alteration of PCSK9 with in vivo CRISPR-Cas9 genome editing. Circ Res.

[94] (2023 Dec 1). First in vivo base editing lowers cholesterol. Nat Biotechnol.

[95] Mahjoubin-Tehran M., Rezaei S., Santos R.D., Jamialahmadi T., Almahmeed W., Sahebkar A. (2024 May 25). Targeting PCSK9 as a key player in lipid metabolism: exploiting the therapeutic and biosensing potential of aptamers. Lipids Health Dis.

[96] Landlinger C., Pouwer M.G., Juno C., van der Hoorn J.W.A., Pieterman E.J., Jukema J.W. (2017 Aug 21). The AT04A vaccine against proprotein convertase subtilisin/kexin type 9 reduces total cholesterol, vascular inflammation, and atherosclerosis in APOE∗3Leiden.CETP mice. Eur Heart J.

[97] Tokgözoğlu L., Pirillo A., Catapano A.L. (2025 Apr 30). Oral PCSK9 inhibitors: will they work?. Curr Atheroscler Rep.

[98] Ferri N., Marodin G. (2024 Dec 12). Emerging oral therapeutic strategies for inhibiting PCSK9. Atheroscler Plus.

[99] Zhang Y., Dai D., Geng S., Rong C., Zou R., Leng X. (2024 Oct). PCSK9 expression in fibrous cap possesses a marker for rupture in advanced plaque. Vasc Med.

[bibnt1] 100The 33 references from the initial search are indicated by an asterisk.

